# Can Larvae of Forest Click Beetles (Coleoptera: Elateridae) Feed on Live Plant Roots?

**DOI:** 10.3390/insects11120850

**Published:** 2020-11-30

**Authors:** Mikhail V. Kozlov, Alexander S. Prosvirov, Elena L. Zvereva

**Affiliations:** 1 Department of Biology, University of Turku, 20014 Turku, Finland; elezve@utu.fi; 2 Department of Entomology, Faculty of Biology, Moscow State University, 119234 Moscow, Russia; carrabus69@mail.ru

**Keywords:** *Athous subfuscus*, *Betula pubescens*, *Dalopius marginatus*, *Deschampsia flexuosa*, feeding strategy, forest pests, *Picea abies*, *Pinus sylvestris*, wireworms

## Abstract

**Simple Summary:**

Detailed natural history information is still lacking for many species of soil-dwelling invertebrates. We asked whether the larvae (wireworms) of two click beetle species, which are most abundant in European boreal forests, feed on live roots of forest plants. The weight of root pieces of downy birch, Scots pine, Norway spruce and wavy hair-grass, offered to wireworms in a laboratory experiment, did not decrease, indicating that these larvae did not consume live root tissues. Therefore, *Athous subfuscus* and *Dalopius marginatus* should be excluded from the lists of forest pests damaging tree roots.

**Abstract:**

The life histories of many soil-dwelling invertebrates remain poorly studied. The larvae of two click beetle species, *Athous subfuscus* and *Dalopius marginatus*, which are most abundant in European boreal forests, are both classified as omnivorous and are included in lists of root-damaging pests. Nevertheless, we are not aware of any direct proof of their ability (or inability) to consume plant roots. In this study, we asked whether these larvae actually feed on the roots of forest plants in the absence of other food sources. Live roots of boreal forest plants, including trees (*Betula pubescens*, *Picea abies* and *Pinus sylvestris*) and grass (*Deschampsia flexuosa*), were offered to click beetle larvae in a two-month microcosm experiment. The weight of roots placed in vials with the wireworms did not decrease, indicating that the larvae of these click beetle species did not feed on live roots, even in the absence of other food sources. This suggests that the feeding niches of *A. subfuscus* and *D. marginatus* larvae are narrower than previously thought and do not include live plant tissues. Therefore, these click beetle species should be excluded from the lists of forest pests damaging tree roots.

## 1. Introduction

The processes occurring in soil are among the most poorly understood areas in ecology [1], although the belowground net primary production (NPP) accounts for 40–70% of total terrestrial productivity [2]. The aboveground plant parts experience an average annual loss of 5–8% of their leaf area to insects [3], and these losses impose substantial adverse effects on tree growth [4,5]. However, the knowledge on belowground NPP consumed by insects and other invertebrates in natural ecosystems is limited to only a few reliable estimates [6,7]. This kind of information is difficult to obtain in field conditions, but it is needed for assessment of the role of belowground herbivory in global carbon and nutrient cycling. Therefore, some studies have attempted to estimate root losses from the biomass of root-feeding insects [8]. However, this approach requires precise knowledge of the feeding habits and feeding specialisations of soil-dwelling organisms, which are uncertain or completely lacking even for some of the most common invertebrates [9,10].

One of the most abundant groups of soil-dwelling insects in boreal forests is represented by the click beetles, which can comprise as much as a quarter of the biomass of the soil macrofauna (M. Kozlov and A. Prosvirov, unpublished data). Several species of click beetles are important agricultural pests, as their larvae attack the roots and tubers of a wide range of crops, including maize and potatoes, both in natural and experimental conditions ([10,11,12,13,14,15,16] and references therein), and their root-feeding habits do not cause any doubts. However, the feeding habits of the larvae of the click beetle species common in boreal forest soils have not been properly documented.

The data that are available on feeding specialisations of click beetle larvae are diverse and often contradictory [10]. For example, the larvae of *Athous subfuscus* (O. F. Müller), the species which dominates click beetle communities in different types of forests [17,18,19], were depicted in several guidebooks on forest pests as damaging to the roots of woody plant seedlings [20,21]. Kula [22] explicitly stated that the larvae of this species “are harmful to the root system of [forest] plants”. At the same time, the larvae of *A. subfuscus* were reported to consume soil organic substances [23] and to feed on a variety of soil animals [24,25,26,27]. The analysis of δ^15^N values suggests that this species is the “most predatory” wireworm among the 11 studied European species [28] but does not exclude the possibility of occasional feeding of *A. subfuscus* larvae on live plant roots. Consequently, different researchers have classified this species as either saprophagous [29], predatory [28,30] or omnivorous—i.e., feeding on a variety of foods of both plant and animal origin [23,27,31]. A similar controversy exists regarding the feeding habits of another common click beetle species, *Dalopius marginatus* (Linnaeus) [10,20,24,26,29,32,33,34]. Nevertheless, we are not aware of any direct proof of the ability (or inability) of *A. subfuscus* and *D. marginatus* to consume plant roots.

The goal of the present study is to experimentally test the hypothesis that the larvae of *A. subfuscus* and *D. marginatus* feed (at least in the absence of animal food and of soil organic substances) on the live fine roots of the most common boreal forest plants.

## 2. Materials and Methods

### 2.1. Study System

Although wireworms have well developed mandibulae, their mouthparts allow consumption of liquids only [35,36]. For example, food particles exceeding 0.003 mm in size could not pass oral filters of *Selatosomus aeripennis* (Kirby) [37]. Therefore, dissection of wireworms cannot be used to visually identify the consumed food, and we tested our hypothesis by assessing the changes in weight of roots offered to larvae.

*Athous subfuscus* and *D. marginatus* are common Palaearctic click beetle species that are widely distributed in Northern and Central Europe [38]. In Scots pine forests of Germany, 75% of wireworms belongs to these two species [25]. Similarly, our earlier experience showed that these two species jointly constitute over 90% of the wireworm community in the forests of southern Finland and northwestern Russia (M. Kozlov and A. Prosvirov, unpublished data). These forests consist either of Scots pine (*Pinus sylvestris*) or Norway spruce (*Picea abies*). Downy birch (*Betula pubescens*) is common in both the pine and the spruce forests. The field-layer vegetation is dominated by bilberry (*Vaccinium myrtillus*). Wavy hair-grass (*Deschampsia flexuosa*) is also a common, although not abundant, component of the forest flora in this region [39].

### 2.2. Experimental Design

We used the experimental methods developed by Kosmatshevsky [13] and Dolin [14]. From 28 June to 5 July 2018, we collected 105 wireworms by hand-sorting soil samples (30 cm in depth) obtained from multiple sites in natural forests around Turku, Helsinki and St. Petersburg. We were unable to control for the species identity of the larvae at the beginning of the experiment, but we identified the larvae at the later stages of our study. We placed wireworms individually in 50 mL plastic vials half-filled with soil from the site of their origin. The individual placement was used to prevent cannibalism, which we frequently observed in these larvae. On 6 July 2018, all larvae were weighed to the nearest 0.1 mg. Larvae with extreme weights were excluded, and 60 larvae with initial live weights ranging from 4.2 to 32.4 mg were individually placed in 50 mL plastic vials. These vials were half-filled with fine crushed stone (grain size ca. 1 mm), which had been carefully washed and then dried at 105 °C. The experiment was conducted at room temperature (ca. +24 °C) and under natural illumination. A sufficient amount of water was added regularly to each vial to keep the substrate wet.

Vials with larvae were randomly assigned to one of three groups (20 vials per group). Larvae from these groups were supplied with live roots of one of three tree species (Scots pine, Norway spruce, or downy birch). Root pieces (diameter 0.2–1.2 mm, length 40–60 mm) were obtained from small (10–30 cm tall) seedlings excavated from the forested site near Turku. These pieces were washed free of soil, weighed (average live weight 25 mg) and then buried at a half-depth in the artificial soil in the vials. Roots from the three tree species were also placed in an additional nine vials (three vials per plant species) without wireworms to control for changes in root biomass during the experiment. We were not interested in the ability of wireworms to survive in the absence of the potential food; therefore, we did not consider larvae which were not supplied with plant roots in our experimental design. After 5 to 7 days of exposure, the roots from each vial were removed, cleaned, examined under stereomicroscope for traces of larval feeding and weighed. Roots from newly excavated plants were weighed and inserted into the vials to replace the original batch of roots.

The larvae of the root-feeding click beetles generally grow well under experimental conditions when provided with sufficient amounts of suitable food—e.g., carrot, potato tubers, non-germinated soybean and corn seeds, and roots of *Lolium multiflorum*, *Medicago sativa*, *Sinapis alba* and *Zea mays* seedlings [13,14,15,16]. Therefore, our experiment was originally planned to run for two to three weeks. However, we found that the first batch of roots removed from the vials showed no decrease in weight, contrary to our expectations. This suggested an absence of root consumption by the wireworms.

Earlier studies had demonstrated that wireworms can cope without food for at least 20 days [13]. Based on this information, we extended our experiment to a two-month duration, assuming that long starvation might force wireworms to consume live roots. Finally, on 27 August, we provided all survivors with roots of wavy hair-grass to test the hypothesis that wireworms feed on roots of herbaceous, rather than woody, forest plants. The experiment was discontinued on 6 September 2018.

We assessed whether each larva was alive during each replacement of roots. All live larvae were weighed on 21 July, and 6, 20 and 27 August; the number of weighed larvae decreased with time, as some larvae died during the course of the experiment. The larvae supplied with grass roots were also weighed on 6 September 2017. All larvae were preserved in 70% ethanol for later identification based on morphological characters. This identification was performed by A.S.P.

### 2.3. Data Analysis

We explored the effects of click beetle species and tree species on wireworm survival time using the Cox proportional hazards model (PHREG procedure [40]). We compared the initial and final weights of the larvae by paired *t* tests. The absolute gain (or loss) of weight by each larva and by each piece of root was calculated as the difference between the initial and final live weights. Changes in weight were analysed by a mixed model ANOVA (GLIMMIX procedure, type III tests [40]). We considered plant species, click beetle species, date of weighing (a repeated factor with compound symmetry as a variance–covariance structure; for larval weight only), treatment (i.e., the presence of wireworms in the vial; for root pieces only) and their interactions as fixed effects, whereas a vial was treated as a random effect. To facilitate accurate *F* tests of the fixed effects, we adjusted the standard errors and denominator degrees of freedom in all our analyses by the latest version of the method described in [41]. The significance of the random factor was evaluated by calculating the likelihood ratio and testing it against the chi-squared distribution [42]. Additionally, we used a one-way ANOVA to compare the initial weight between the larvae which died during the experiment and which survived until the end of it, and to compare the relative changes in larval weight during the experiment between small (initial weight 4.5–10.2 mg; n = 11) and large (initial weight 10.3–22.6 mg; n = 11) larvae.

## 3. Results

The larvae selected for the experiment belonged to four click beetle species: *A. subfuscus* (41 larvae), *D. marginatus* (four larvae), *Ampedus balteatus* (Linnaeus) (two larvae) and *Mosotalesus impressus* (Fabricius) (one larva). Identification of the remaining 12 larvae was not possible due to their deterioration during the course of the experiment.

**Figure 1:**
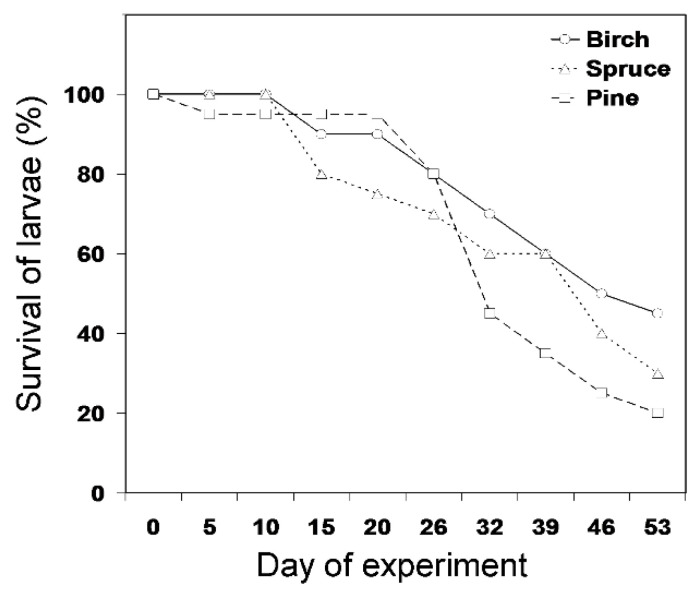
Survival of wireworms supplied with roots of different tree species. The sample size at the start of the experiment was 20 wireworms per tree species. For statistical analysis, see the main text.

The survival time did not differ among the click beetle species (Wald *χ*^2^ = 1.35, df = 3, *p* = 0.71) or among the wireworms supplied with roots of different tree species (Figure 1; *χ*^2^ = 3.39, df = 2, *p* = 0.18). The latter result did not change when the analysis was restricted to the most abundant species, *A. subfuscus* (*χ*^2^ = 3.21, df = 2, *p* = 0.20). About one-quarter of our larvae moulted during the experiment, but the moulting was not synchronised among individuals. The initial weight did not differ between the larvae that died during the experiment and those that survived until the end of it (*F*_1, 58_ = 2.50, *p* = 0.12).

**Figure 2:**
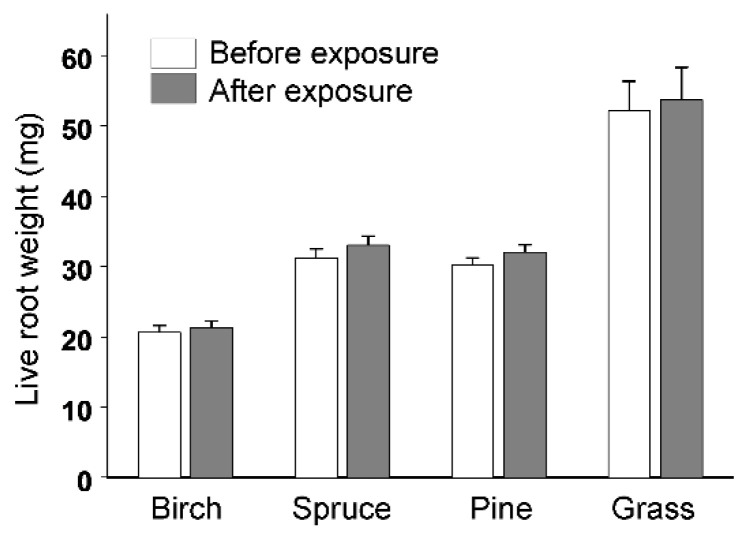
Live root weight (means + S.E.) of forest plants before and after 5 to 7 days of exposure in vials containing wireworms. Sample sizes: birch, 143; spruce, 132; pine, 137; grass, 10. For statistical analysis, see the main text.

The weight of the root pieces placed in vials with wireworms (Figure 2) increased slightly but significantly (mean ± S.E.: 1.34 ± 0.09 mg; paired test: *t*_409_ = 14.2, *p* < 0.0001) during the time of their exposure. The differences in weight of the root pieces before and after their exposure did not differ between vials with and without wireworms (*F*_1, 490_ = 0.02, *p* = 0.89), and the microscopic examination did not reveal any traces of mandibles on roots that have been exposed to wireworms. The live weights of wireworms did not change between the subsequent weightings (Table 1). However, 14 larvae of *A. subfuscus* that were alive at the end of the experiment had gained, on average, 3.65 ± 1.26 mg of fresh weight (27.5 ± 6.6% of their initial weight) between 6 July and 27 August (paired test; *t*_13_ = 2.90, *p* = 0.01). The larvae that were larger at the beginning of the experiment tended to gain more weight relative to the smaller larvae (*F*_1, 12_ = 3.62, *p* = 0.08).

## 4. Discussion

Our experiment demonstrated that none of the larvae of any of the four studied click beetle species (*A. subfuscus*, *D. marginatus*, *A. balteatus* and *M. impressus*) consumed live fine roots of forest plants. This was true for both woody and herbaceous species, as indicated by the lack of biomass loss in the roots provided as the sole food for these larvae. Even a long period of starvation did not force these wireworms to start feeding on the supplied live roots. The observed increases in live weight of the wireworms during our experiment may reflect an absorption of water, as water absorption was found to increase their weight by 20–30% in earlier experiments [43]. Similarly, the slight increase in weight of the root pieces offered to the wireworms was also likely due to water absorption. There also exists a possibility that larvae consumed microbial (especially fungal) biomass introduced with plant roots to the artificial ground [44], but this possibility does not compromise our conclusion on the absence of root consumption.

Our results clearly contrast with the published information regarding the feeding by the larvae of these click beetle species on plant roots. In our opinion, the discrepancy between our results and those in earlier publications may have resulted from (i) erroneous association of wireworms with root damage imposed by other organisms, (ii) erroneous identification of click beetle larvae as the organisms damaging forest plant roots, (iii) uncritical use of fragmentary observational data and/or (iv) unjustified generalisation of natural history information.

The first of these possibilities was carefully discussed by Escherich [45], and we have little to add to his comments regarding the need for cautious attribution of the observed root damage to one of the soil-dwelling organisms. Importantly, the densities of *A. subfuscus* larvae can be very high in forest soils (200‒300 larvae m^−2^ [18]). Therefore, these larvae might easily be assumed to be the immediate cause of any observed root damage, especially when wireworms prey on insects that make wounds in thick roots. However, in our opinion, the most probable source of the information regarding root damage inflicted by *A. subfuscus* and *D. marginatus* larvae is misidentification, as this frequently occurs in publications on plant pests [46].

The likely primary source of the problematic information regarding root feeding by larvae of *D. marginatus* and *A. subfuscus* was published 140 years ago. Altum [47] illustrated thick roots damaged by *D. marginatus* larvae and wrote that these wireworms were stuck inside the root wounds. However, he did not provide any proof that these wounds were made by wireworms or that the wounded root tissues were even live at the time of damage. Altum [47] also mentioned that some of larvae, which he considered to be root-damaging types, belonged to the group that includes *A. subfuscus* and *Selatosomus aeneus* (Linnaeus). A few years later, Beling [48] clearly stated that larvae of *D. marginatus* and *A. subfuscus* could damage roots and/or seeds of different forest plants.

These (or similar) occasional observations could have resulted in the attribution of *D. marginatus* and *A. subfuscus* as pests of plant roots in subsequent publications, due, in particular, to inaccurate citation of earlier works. For example, Pavlovskii and Shtakelberg [20] refer to Escherich [45] and Sorauer [49] when stating that *A. subfuscus* larvae feed on roots of forest trees. However, Escherich [45] did not mention *A. subfuscus* among the species damaging to roots of tree seedlings, and Sorauer [49] only wrote that larvae of this species, in the absence of animal food, can damage the seeds of several forest plants. This statement was repeated by Horion [50], although he mentioned that both *D. marginatus* and *A. subfuscus* prey on larvae and pupae of forest pests [25,26].

Last but not least, the authors of guidebooks may have incorrectly generalised the knowledge obtained for one species to a wider group of species, while paying little attention to among-species differences in feeding habits, despite the fact that these differences can be very strong even between closely related click beetle species. For example, the larvae of different *Athous* species show feeding habits ranging from phytophagy, where they eat live plants among other foods, through accidental phytophagy to obligatory predation [14]. Nevertheless, some guides [51,52] include the genus *Athous*, as a whole, in the list of pests damaging to plant roots.

## 5. Conclusions

We appreciate that our study has several limitations, including relatively small sample size for *D. marginatus*. Nevertheless, our results clearly indicate that the larvae of *A. subfuscus* and *D. marginatus* do not feed on live roots of plants, which are common in the coniferous forests of Northern Europe. At least for this region, these click beetle species should be removed from the lists of pests that damage the roots of forest trees. Our findings demonstrate that our knowledge of the feeding strategies of even the most abundant soil-dwelling invertebrates is still imperfect. Biological data published long ago, even those that have been repeatedly cited in subsequent publications, should be used with caution, because they may be erroneous.

## Figures and Tables

**Table 1:** Sources of variation in live weight of wireworms supplied by roots of downy birch, Scots pine or Norway spruce (repeated linear mixed model: GLIMMIX procedure, type III tests) during two months of microcosm experiment.

Effect	Explanatory Variable	Test Statistics	*p* Value
Fixed	Click beetle species (BS)	*F*_3, 40.1_ = 1.93	0.14
	Tree species (TS)	*F*_2, 42.4_ = 0.06	0.94
	Date of weighing (DW)	*F*_4, 89.2_ = 1.12	0.35
	BS × TS	*F*_1, 39.5_ = 0.09	0.76
	BS × DW	*F*_1, 89.4_ = 0.74	0.69
	TS × DW	*F*_8, 89.8_ = 0.53	0.83
	BS × TS × DW	*F*_4, 89.3_ = 0.21	0.93
Random	Individual larva	*χ*^2^ = 144.2	<0.0001
